# Breast Cancer Treatment Strategies Targeting the Tumor Microenvironment: How to Convert “Cold” Tumors to “Hot” Tumors

**DOI:** 10.3390/ijms25137208

**Published:** 2024-06-29

**Authors:** Liucui Yang, Qingyi Hu, Tao Huang

**Affiliations:** Department of Breast and Thyroid Surgery, Union Hospital, Tongji Medical College, Huazhong University of Science and Technology, Wuhan 430030, China; ieonglaochoi@gmail.com

**Keywords:** tumor microenvironment, breast cancer, immunotherapy, nanoparticles, nano-immunotherapy, target therapy, novel agents

## Abstract

Breast cancer characterized as “cold tumors” exhibit low levels of immune cell infiltration, which limits the efficacy of conventional immunotherapy. Recent studies have focused on strategies using nanotechnology combined with tumor microenvironment modulation to transform “cold tumors” into “hot tumors”. This approach involves the use of functionalized nanoparticles that target and modify the tumor microenvironment to promote the infiltration and activation of antitumor immune cells. By delivering immune activators or blocking immunosuppressive signals, these nanoparticles activate otherwise dormant immune responses, enhancing tumor immunogenicity and the therapeutic response. These strategies not only promise to increase the response rate of breast cancer patients to existing immunotherapies but also may pave new therapeutic avenues, providing a new direction for the immunotherapy of breast cancer.

## 1. Introduction

In the past decade (2010–2019), the incidence of breast cancer (BC) has gradually increased at a rate of 0.5% per year [1]. In 2020, female BC became the most commonly diagnosed cancer in the world for the first time, with an estimated number of new cases reaching 2.26 million [2]. By 2040, the global burden of BC is expected to increase to more than 3 million new cases per year [3]. Surgical treatment, radiotherapy and chemotherapy remain the basic methods for treating BC. For early-stage patients (stage I or II), surgical treatment can be combined with postoperative chemoradiotherapy; for stage III BC, patients mainly receive chemoradiotherapy, and a few receive mastectomy; for distant metastasis (stage IV) BC, patients only receive chemoradiotherapy, and hormone receptor (HR)-positive patients can choose endocrine therapy. The five-year relative survival rates for BC differ by stage. Stage I has a 99% survival rate, with the cancer confined solely to the breast. Stage II shows a survival rate of 93–97%, as the cancer spreads to adjacent lymph nodes. In Stage III, the rate is 72–86% when the cancer reaches distant lymph nodes or muscles. Stage IV, or metastatic BC, has a survival rate of about 28%, with cancer spreading to distant sites. These data emphasize the critical challenges in treating BC, especially late-stage (stage III or IV) [4].

Triple-negative breast cancer (TNBC) is a highly aggressive subtype of BC that is associated with a poor prognosis, high recurrence and metastasis rates [5]. Neoadjuvant chemotherapy is the primary treatment option for early-stage TNBC due to the absence of available endocrine or targeted therapies [6]. However, neoadjuvant therapy achieved pathological complete remission (pCR) in 30–40% of TNBC patients. The remaining patients showed moderate response or drug resistance [7].

**Figure 1:**
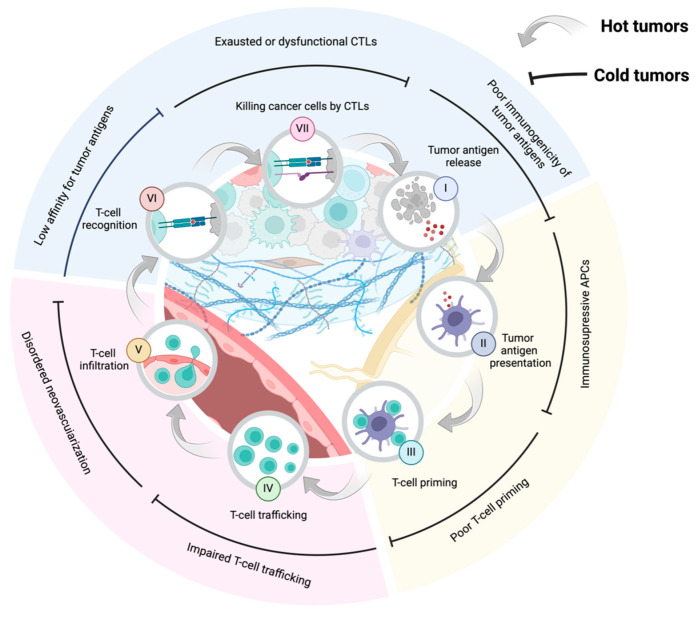
Transforming cold tumors into hot tumors by modulating the cancer–immunity cycle. The cancer–immunity cycle starts with the release of cancer cell antigens (I), followed by their presentation to T cells by dendritic cells (II). This leads to T cells activation and their migration to the tumor site, where they infiltrate, recognize and kill the cancer cells (III–VII). The destruction of cancer cells releases new antigens, perpetuating the cycle and sustaining the immune response against the tumor (VII–I). Each step in this cycle offers potential targets for enhancing immunotherapy treatments.

Conventional clinical interventions frequently result in the destruction of healthy tissues and increase the risk of tumor recurrence [8]. In contrast, immunotherapy is a more effective and specific treatment for tumor recognition by enhancing immune surveillance. As an innate immune “cold” tumor, in BC processes of occurrence and development, cancer cells reduce immunogenicity to evade the host immune surveillance, inhibit intratumoral infiltration and the activation of cytotoxic T lymphocytes (CTLs) [9,10]. On the other hand, there are a large number of tumors infiltrating immune cells in the immunosuppressive tumor microenvironment (TME) of BC, which promote tumor progression by promoting cancer cells proliferation, vascular formation and immunosuppression (Figure 1) [11,12]. Up until now, the results in clinical trials of immunotherapy have been disappointing. Nevertheless, the modulation of the immunosuppressive TME to convert “cold” immunosuppression tumors into “hot” immune-activating tumors represents a promising avenue for enhancing the efficacy of BC treatment [13].

## 2. Tumor Microenvironment, the “Soil” on Which Cancer Cells Depend for Survival

In the initial stages of cancer development, immune cells and cancer cells collaboratively establish the TME to foster tumor plasticity and heterogeneity. The recognition of the TME’s role in cancer emerged as Hanahan and Weinberg outlined ten biological hallmarks that characterize cancer complexity [14,15,16]. These include proliferative signaling, growth suppressor evasion, cell death resistance, angiogenesis, metastasis activation, replicative immortality, metabolic reprogramming, immune evasion, inflammation promotion and genome instability. This framework enhances our understanding of cancer and drives the development of targeted therapies, advancing oncology significantly.

In fact, the TME contributes to various aspects in promoting cancer occurrence, which include initiation, survival, growth, metastasis and immune evasion. The previous cancer researchers mainly focused on the tumor cell itself and a series of molecular events occurring within the tumor cell, limiting cancer research to the cancer cell itself, ignoring the role of the TME and the systemic regulation of the body in the occurrence and development of cancer, which is very one-sided.

Based on the different immune components, the TME is classified into “cold” and “hot” phenotypes [17]. Immune “cold” tumors are characterized by low tumor immunogenicity, defects in tumor antigen processing and presentation mechanisms, insufficient T cells infiltration and an immunosuppressive TME [18]. These features significantly impact the effectiveness of immunotherapies and the overall prognosis of cancer patients. Although some immune populations in TME have the potential to clear malignant cells, many factors in tumors can weaken this activity or reprogram cells to promote tumorigenesis. For example, dendritic cells (DCs) and macrophages are the most important antigen-presenting cells (APCs) in the TME. Meanwhile, tumor-associated macrophage (TAM) polarization, increased myeloid-derived suppressor cells (MDSCs) and DC maturation inhibition can exacerbate immunosuppressive TME that jointly coordinates the activation and function of antitumor CD8^+^ T cells [19,20,21].

In addition to the relative proportion, the location of immune cells is also associated with disease progression. For instance, in ductal carcinoma in situ (DCIS), focal dysplasia of the myoepithelial cell layer is associated with leukocyte enrichment, suggesting its role in promoting or sustaining local invasion [22]. Additionally, T cells rejection within the tumor niche may enhance resistance to immune checkpoint inhibitor (ICI) therapy in advanced stages of the disease [23,24].

Non-immune stromal cells, including fibroblasts, endothelial cells and pericytes, also exhibit unique gene/protein expression profiles distinct from their normal tissue counterparts and contribute to the characteristics of a “cold” TME. For example, cancer-associated fibroblasts (CAFs) can suppress T cells responses through various pathways. They directly inhibit T cells by expressing PD-L2 and FAS ligands and indirectly by secreting cytokines and recruiting immunosuppressive myeloid cells [25,26,27].

The physical and chemical properties of the TME, such as matrix stiffness, density, composition, pH value and interstitial fluid flow, are dysregulated, impacting cancer risk, tumor progression and treatment efficacy [28,29]. The hardness of most solid tumors, including BC, is significantly higher compared to surrounding normal tissues, and they exhibit a fibrotic state known as the “desmoplastic reaction” [30,31]. The alterations in the physical properties of the matrix further regulate the TME [32], such as higher levels of collagen and collagen crosslinking, which enhance pro-tumorigenic phenotypic macrophages [33,34,35]. The uncontrolled proliferation of cancer cells leads to poor hemoperfusion efficiency and increased glucose metabolism rates, forming an acidic TME. Decreased pH values are associated with tumor aggressiveness, angiogenesis, cell clock disruption and stemness, which also hinder CD8^+^ T cells reactivity [36,37,38,39,40].

Our understanding of the complex interaction between cancer and the immune system has shifted from the concept of “immune surveillance” to the concept of “immune editing”, which brings great hope for the treatment of malignant tumors by editorializing the immune microenvironment to monitor and control tumor development.

## 3. Strategies of Editing the Tumor Microenvironment for Breast Cancer Treatment: Nano-Based Approaches

### 3.1. T Cells

T cells, as essential effector cells, execute a vital function in antitumor immunity. The abundant infiltration of T cells is also a hallmark characteristic of “hot tumors”. Consequently, the strategies for recruiting and augmenting the activity of T cells within the TME have emerged as the central focus of researchers [41,42,43,44].

T cell bispecific antibodies (TCBs) are characterized by their dual antigen-binding domains derived from two distinct proteins [45]. Each domain is specifically designed to engage with different cellular targets—one domain targets T cells, while the other is directed against tumor cells. For instance, TCBs employ antibodies against CD3, a marker on T cells, in conjunction with antibodies that recognize specific tumor cell antigens. This design facilitates the redirection of CD3-positive T cells towards tumor cells, enabling a focused and potent immune-mediated attack on the cancer cells. Owing to the overexpression of human epidermal growth factor receptor 2 (HER2) in approximately 20% of primary invasive BCs, CD3/HER2 TCB is extensively employed in clinical studies. Nonetheless, severe cases of targeted toxicity have been observed in clinical trials (NCT00924287) [46,47,48]. To avoid side effects, attempts are being made to confine the selection of targeted antigens to those that are exclusively expressed in tumor cells, such as p95HER2, which present in merely 40% of HER2-positive BCs. Alternatively, the targeting of bispecific antibodies necessitates enhancement to avoid off-targeting [49].

TNBC demonstrates no overexpression of HER2, and CD3/TROP2 TCB (F7AK3) exhibits specific binding to both T cells and TROP2, which is overexpressed in TNBC cell lines such as MDA-MB-231, MDA-MB-468 and HCC1395. In animal studies, F7AK3 has been observed to facilitate a significant expansion of immune cells within tumors and the spleen, elevate IL-2 expression and reduce tumor size and growth rate (Table 1) [50].

The proliferation, survival and differentiation of T cells are primarily governed by interleukin (IL)-like cytokines, such as IL-2, IL-12 and IL-15. Initial attempts focused on augmenting T cells responses through the systemic administration of free cytokines, resulting in tumor rejection and an antitumor memory immune response, without inducing an increase in generating Tregs [71,72,73]. Yet, these approaches were soon abandoned due to their toxicity and limited half-times. 

Nanoparticles (NPs) can deliver immune modulators directly to T cells, enhancing their activation and proliferation. The Enhanced Permeability and Retention (EPR) effect aids NP accumulation in tumor tissues. Functionalized NPs with specific ligands target T cells receptors for enhanced uptake [74]. A study demonstrated that immunostimulatory NPs reprogrammed dysfunctional APCs, leading to a significant activation of cytotoxic T cells via robust interferon-β production. This approach improved both short-term and long-term immune responses in breast cancer, particularly in the 4T1 mouse model [75].

### 3.2. Tumor-associated Macrophages, TAMs

**Figure 2:**
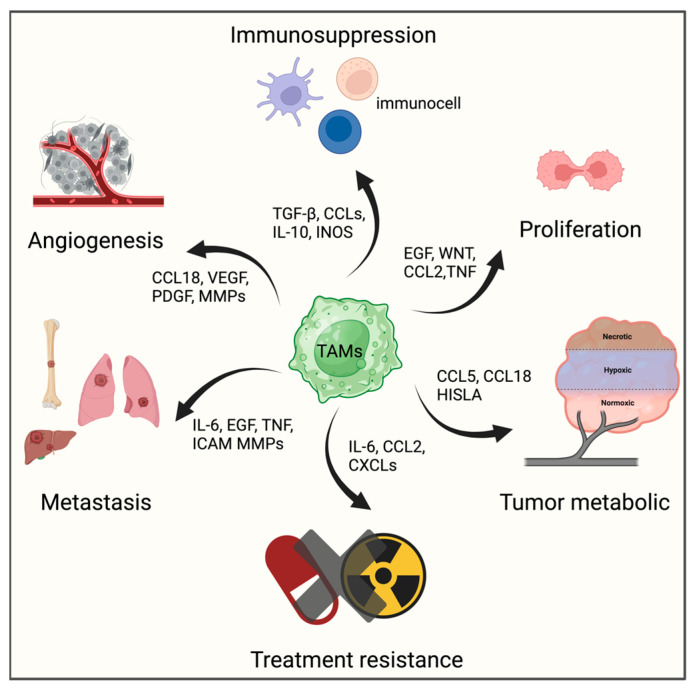
The biological functions and secreted cytokines of tumor-associated macrophages (TAMs). (1) Angiogenesis: TAMs promote the formation of new blood vessels, which supply tumors with oxygen and nutrients. (2) Immunosuppression: TAMs suppress immune responses against tumors by producing immunosuppressive cytokines. (3) Cell Survival and Proliferation: TAMs release growth factors that enhance tumor cell survival and proliferation. (4) Tumor Metabolism: TAMs adapt the tumor metabolism for rapid growth by enhancing the nutrient availability and adapting to low oxygen. (5) Treatment Resistance: TAMs promote resistance by altering the tumor environment and reducing the effectiveness of therapies. (6) Metastasis: TAMs assist in cancer metastasis by degrading extracellular matrices, facilitating the tumor cell invasion of other tissues.

Macrophages, as the most abundant immune cell type, are prevalent in various solid tumors, including BC. Tumor-infiltrating macrophages predominantly exhibit the M2 phenotype, instigating pro-tumor activities such as tumor progression, angiogenesis and metastasis, also known as tumor-associated macrophages (TAMs) [76,77,78,79,80,81,82,83]. Moreover, they are capable of secreting immunosuppressive cytokines and enzymes or presenting immune checkpoint proteins (notably PD-L1), which cause CD8^+^ T cells dysfunction, prompting in the generation of immunosuppressive TME (Figure 2) [84,85].

The initiation of M2 polarization signaling requires the binding of colony-stimulating factor (M-CSF) to the CSF1 receptor on macrophage surfaces. Additionally, the interaction between tumor cell CD47 and macrophage SIRPa inhibits the phagocytic function of the macrophages [86]. The development of tumors was effectively suppressed, and the population of M1-polarized macrophages was notably augmented in animal tests through the application of either small molecule inhibitors or nanoparticle-supported inhibitors. Nonetheless, the observed therapeutic outcome failed to meet the anticipated expectations [87]. NPs have emerged as a crucial element in facilitating multimodal therapy, enabling the delivery of potent drugs such as Doxorubicin and Paclitaxel to eradicate cancer cells and modulate macrophages (Table 2) [88,89,90].

Paclitaxel, for instance, has been demonstrated to reprogram M2-polarized macrophages into a M1-like phenotype via a TLR4-dependent pathway, thereby enhancing the antitumor immunity. In both in vitro and in vivo models, Paclitaxel not only inhibits tumor cell growth but also shifts the TAM profile from a M2-like to a M1-like phenotype. This process is potentially mediated through the activation of the TLR4 receptor and associated changes in gene expression and the release of proinflammatory cytokines [88]. Moreover, Paclitaxel can activate the cGAS-STING signaling pathway, prompting tumor cells to release soluble factors that induce M1-like polarization in macrophages [101]. In addition, a study on Paclitaxel demonstrated its ability to suppress the induction of M2 macrophages, suggesting a shift towards an M1-like profile even at lower concentrations [102].

However, numerous medications that influence macrophages also impact other immune cell populations. Thus, determining the authentic mechanism for macrophage-specific nano-immunotherapy remains a daunting task. Comprehensive immunoanalyses conducted at varying times within the tumor, lymph node/spleen and other relevant locations may provide new knowledge.

### 3.3. Dendritic Cells, DCs

**Figure 3:**
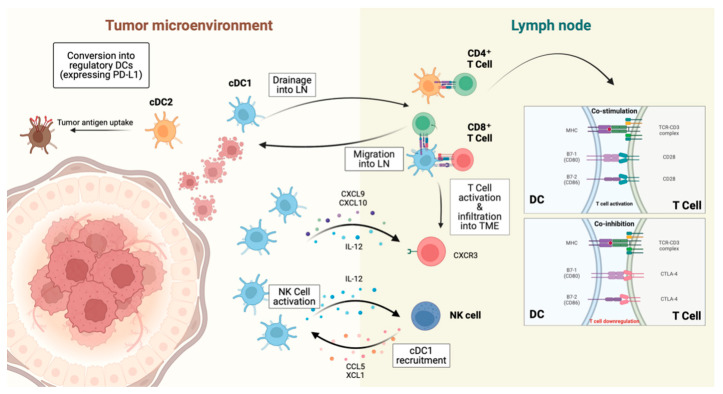
The immune regulation of DC in tumor microenvironment. cDCs migrate to lymph nodes to prime T cells: cDC1s activate both CD4^+^ and CD8^+^ T cells, while cDC2s primarily activate CD4^+^ T cells. cDC1s produce CXCL9 and CXCL10, attracting activated T cells via CXCR3. DCs also regulate T cells activation through various co-stimulatory molecules. Interaction with NK cells enhances this dynamic, with NK cells promoting cDC1 recruitment and activation via chemokines like CCL5 and XCL1 and cDC1s, in turn, boosting NK cell function with IL-12. After antigen uptake, cDCs can become regulatory DCs expressing PD-L1, modulating immune responses towards either suppression or tolerance.

DCs are crucial for generating long-lasting antitumor immunity by activating CTLs and NK cells (Figure 3) [103]. However, within the immunosuppressive TME, DCs often remain immature and tolerant [104]. DC vaccination can reverse this state, enhancing CTL and NK cell activation, increasing effector immune cell infiltration and reducing immunosuppressive cells like Tregs and MDSCs. Combining DC vaccination with therapies such as ICIs has shown synergistic effects, improving treatment outcomes and extending the progression-free survival in BC patients.

NPs enhance DC functions by delivering antigens or adjuvants (Table 3). They can penetrate dense tumor stroma and are functionalized with ligands targeting DC-specific receptors. In vitro methods such as culture medium incubation and electroporation can effectively introduce NPs into DCs. In vivo methods, like local injection and intravenous injection, rely on the natural immune surveillance function of DCs to uptake the NPs. The properties of NPs, such as size, shape, surface charge and coating, significantly influence their uptake efficiency by DCs. It is crucial to ensure that the DCs’ viability and functionality are not compromised during the loading process, as they need to retain their ability to process and present antigens to T cells to activate an immune response [105].

AIRISE-02, a NP loaded with CpG and siRNA targeting STAT3, enhances the immune response and inhibits the immunosuppressive TME. After intratumoral injection into tumors like melanoma and breast tumors, CpG stimulates local APCs, especially DCs, while si-STAT3 eliminates immunosuppressive pathways in cancer and bone marrow cells. The combined application of AIRISE-02 with ICIs has shown significantly better therapeutic effects than ICIs alone. Additionally, AIRISE-02 is effective in inhibiting tumor growth after intravenous injection and is currently entering the investigational new drug (IND) phase [114,115,116,117,118].

Overall, optimizing the TME and using DC vaccination with nanotechnology to activate antitumor immune responses are promising strategies for enhancing breast cancer therapies. Nanotechnology facilitates the targeted delivery of immunomodulatory agents, improving the maturation and function of dendritic cells within the TME. 

### 3.4. Cancer-Associated Fibroblasts, CAFs

**Figure 4:**
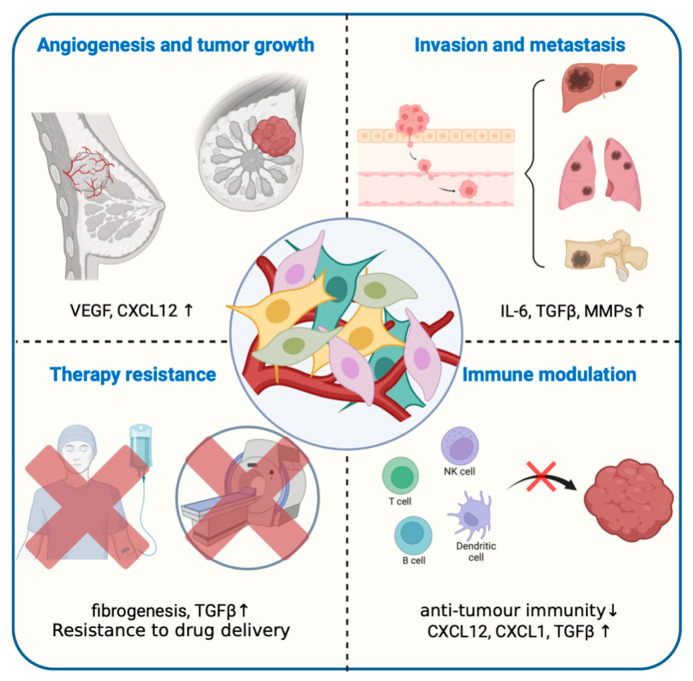
Diagrammatic illustration of selected pro-tumorigenic roles of cancer-associated fibroblasts (CAFs).

The stromal cells in the TME include vascular endothelial cells, pericytes, adipocytes, fibroblasts and bone marrow mesenchymal stromal cells. CAFs mediate fibroproliferative responses and serve as principal instigators of immunosuppressive microenvironments, leading to the failure of immunotherapy in solid tumors (Figure 4) [119,120]. The consumption of CAFs may enhance the efficacy of immunotherapy, which is currently under extensive investigation. Recent research has indicated that TGF-β inhibitors are capable of inhibiting the development of CAFs. For instance, the release of TGF-β inhibitors driven by heparanase (HPSE) at extracellular sites prevents tumor spreading by changing the TME, reducing CAFs activation and minimizing TGF-β production [121,122]. Inducing quiescence in CAFs through the use of angiotensin receptor blockers (ARBs) [123], such as valsartan, may prove to be a more effective strategy than depleting CAFs, which can potentially enhance tumor progression by promoting metastasis. The conjugation of Valsartan with a polyacetal polymer then precipitated into NPs, yielding the preparation of ARB NPs that were activated only in the TME. This approach effectively avoided the systemic adverse reactions induced by the ARB’s involvement in various physiological processes (Table 4).

Nanomedicines can modulate CAF activity and reduce the extracellular matrix (ECM) components to enhance the permeability of solid tumors to therapeutic agents and immune cells. At the tissue level, NPs are designed to penetrate the dense ECM. At the cellular level, they are functionalized with ligands that target fibroblast activation protein (FAP) on CAFs. This approach aims to compromise the tumor integrity and increase the treatment effectiveness, and the current research should focus on modulating CAF functions rather than depleting these cells to avoid increasing tumor aggressiveness.

### 3.5. Tumor-Associated Neutrophils, TANs

Neutrophils, known for their role in inflammation and immunity, display both tumor-promoting and tumor-suppressing functions. The antitumor N1 TANs enhance cytotoxic T cells responses, while the pro-tumor N2 TANs promote angiogenesis and suppress T cells. Targeting chemokines like CXCL1/2 can reduce pro-tumor neutrophils. Agents like TGF-β inhibitors can shift neutrophils to an antitumor phenotype, enhancing immunotherapy [131].

NPs loaded with TGF-β inhibitors can modulate neutrophils within tumors, enhancing local immune responses while minimizing systemic side effects. Furthermore, inhibiting neutrophil extracellular traps (NETs) with agents like DNase I improves PD-1 blockade immunotherapy in TNBC, enhancing CD8^+^ T cells infiltration and activity, thus overcoming resistance and achieving durable clinical responses [132,133].

### 3.6. ErbB/HER Signaling

In the molecular characteristics of BC, besides the amplification of the Her2/neu gene being associated with invasive BC and unfavorable prognosis, Her3 and Her4 belong to the ErbB receptor family and interact with Her2 to play a critical role in signal transduction, cell proliferation and survival in BC. Integrating these biomarkers into BC treatment strategies could lead to more precise and effective therapies [134].

Combining DC vaccination with agents targeting Her3 and Her4 may synergistically enhance antitumor immune responses, thereby improving outcomes. Bispecific antibodies targeting Her2 and Her3, or Her2 and Her4, are being developed to more effectively inhibit these signaling networks [135,136]. Furthermore, inhibitors of downstream pathways activated by Her3 and Her4, such as PI3K/Akt and MAPK, can be combined with these therapies to overcome resistance mechanisms [137]. 

### 3.7. Physical and Chemical Properties

The rapid proliferation of cancer cells causes glycolysis, resulting in an acidic pH at the tumor site. In the TME, the acidic pH allows the NPs to take advantage of the difference in pH between normal cells and cancer cells to target the tumor and achieve a controlled release of pH sensitivity, optimizing the biological distribution of the tumor site and reducing the therapeutic side effects [138,139]. Moreover, various activation technologies are used to enhance the efficacy of NPs conjugated with antitumor agents. For example, magnetic fields can induce temperature changes in lipids, facilitating magnetically triggered hyperthermia stimulation [140]. These magnetic liposomes have demonstrated significant antitumor activity against MDA-MB-231 cells [141].

In the TME, factors such as inadequate blood flow, elevated interstitial fluid pressure, heightened vascularity and interstitial obstruction have a pronounced impact on the permeability and passive targeting of drugs, inhibiting the antitumor efficacy. The enzyme-based NP drug delivery system uses different enzymes, including collagenase, hyaluronidase and matrix metalloproteinases, to degrade dense extracellular mechanisms [142,143]. This enhances the permeability of therapeutic drugs and NPs, enabling effective cancer targeted therapy by improving their access to tumor tissues. For example, AuNPs have shown the highest bioavailability and lowest cytotoxicity among various metals [144]. Gold NPs possess excellent biocompatibility and can be easily functionalized for targeted delivery, making them ideal for drug delivery and imaging applications [145].

## 4. Conclusions and Prospects

With the deepening of the overall understanding of tumors, it has been widely recognized that traditional oncogene therapy is insufficient to treat all patients. Combining or exclusively targeting immune cells and other components within the tumor microenvironment, by enhancing the body’s own immune response and promoting the antitumor effects of various components, can help improve the current treatment landscape and benefit the majority of cancer patients.

The targeted delivery of drugs to exert their effects is a crucial step in antitumor therapy. Efficiently delivering antitumor drugs to tumor tissues through the bloodstream has become a significant focus of new therapeutic strategies. However, the rapid clearance of nanomaterials by the reticuloendothelial system (RES) remains a critical challenge. Stealth nanocarriers, often modified with polyethylene glycol (PEG), aim to evade immune detection but still experience significant early clearance, leading to reduced efficacy. Pseudo-stealth nanocarriers, which exhibit partial stealth effects, offer some improvements but require further optimization. Future research should prioritize the engineering of nanoparticles to enhance their stealth properties, prolong the circulation time and improve bioavailability. Strategies such as optimizing surface coatings, exploring pathogen-mimetic approaches and adjusting physical characteristics like stiffness are essential to overcome RES-related challenges and maximize the therapeutic potential of nanomedicine in cancer treatment [146].

NP delivery systems offer several general advantages within the therapeutic landscape, which include (1) the co-delivery of multiple drugs: Different physicochemical properties of drugs can be accommodated by designing NPs with corresponding characteristics to achieve combination therapy [147,148]; (2) precise targeting: The EPR effect or ligand (e.g., antibodies or peptides) modification of NPs facilitates the accumulation of drug-loaded NPs in tumor tissues [149]; and (3) evasion of clearance: Modifying the external coating of NPs can reduce their recognition and clearance by the mononuclear phagocyte system (MPS) or enzymatic degradation. Additionally, NPs can reduce the required dosage of therapeutic agents, thereby decreasing the antitumor side effects and promoting immune responses. Immuno-oncological nanomedicine expands potential targets to the entire immune system or the overall ecosystem of the tumor host. During treatment, the synergistic work of each part of the innate and adaptive immunity influences the therapeutic efficacy. Increasing efforts are attempting to use combination therapies to mobilize different parts of the body, thereby improving treatment outcomes. Tumor nanotherapy combined with immunotherapy helps weaken the biological barrier effects of tumors and activates immune cells in small portions of the tumor or peripheral tissues to propagate the immune response, thereby more effectively enhancing targeted tumor cytotoxicity. Consequently, the demand for NPs in cancer treatment is increasing due to their exceptional performance in various aspects [150]. By integrating advanced genetic and immunotherapeutic strategies, NPs have the potential to achieve more efficient and precise tumor targeting and treatment, ultimately benefiting a broader range of cancer patients.

NPs are currently in various stages of development, with some already in clinical trials or approved for use. However, comprehensive studies on the toxicology or potential immune responses are still needed to ensure their safety and efficacy. This limitation is particularly evident in the evaluation of immunotherapy for BC, where there is a significant deficiency in tumor models. The most commonly used models are the 4T1 tumor cell model, spontaneous tumor models and patient-derived xenograft (PDX) models [151,152]. Despite their utility, these models have inherent limitations due to issues such as tumor heterogeneity and patient-specific differences, making it challenging to effectively demonstrate the safety and efficacy of nanotechnology-based immunotherapy. Therefore, while clinical advancements in NPs are promising, ongoing research and development are essential to address these challenges and optimize their application in cancer therapy.

Organoids, an emerging three-dimensional cell culture technology, have been shown to faithfully replicate the actual conditions of patients. BC organoids have been utilized for cocultures with various immune cells, creating a system that more accurately simulates the TME. This system is used to study cancer–immune interactions and evaluate the therapeutic efficacy. Research has demonstrated that NPs can effectively penetrate tumor 3D models, deliver antitumor drugs, activate immune cells and enhance the therapeutic efficacy [153].

Due to the heterogeneity (including intertumor and intratumor variations) of breast tumors, accurately predicting the targeting characteristics is challenging in clinical practice. Therefore, the modularity of enhanced therapeutic strategies can help fine tune formulations, allowing for formulation adjustments between individual patients in the treatment process to meet individualized needs.

## Figures and Tables

**Table 1:** Cancer antigens and therapeutic bispecific antibodies of the treatment of breast cancer.

Cancer Antigen	Antibody Targets	References
HER2	CD3 × HER2 mPEG × HER2 CD47 × HER2	[51,52,53]
HER3	CD3 × HER3 HER2 × HER3 EFGR × HER3	[51,54,55]
p95HER2	CD3 × p95HER2	[56]
Trop-2	CD3 × Trop-2	[57]
CEACAM-5	CD3 × CEACAM-5	[57]
EphA10	CD3 × EphA10	[58]
EpCAM	CD3 × EpCAM	[59,60]
p-Cadherin	CD3 × p-Cadherin	[61]
EGFR	CD3 × EGFR mPEG × EGFR	[62,63]
Notch	EFGR × notch	[64]
Mesothelin	CD16 × mesothelin	[65]
Muc1	CD16 × Muc1	[66]
CTLA-4	PD-1 × CTLA-4	[67]
LAG-3	PD-1 × LAG-3	[68]
TGFβ	PD-L1 × TGFβ	[69,70]

**Table 2:** TAMs-mediated nanoparticles against breast cancer.

Payloads or Associated Agents	Nanocarrier	Function	References
Doxorubicin	cleavable PEG chains covering the folate–modified liposome	target tumor cells and M2-TAMs; induce ICD at tumor sites; activate effector T cells (combined with CpG immune adjuvant); reduce M2-TAMs; promote maturation of DCs;	[91]
Doxorubicin	poly(lactic-co-glycolic) acid NPs functionalized by acid-sensitive sheddable PEGylation and mannose modification	reduce TAM population and density in tumor tissues	[92]
Doxorubicin, mitomycin C	iRGD peptide (internalizing Arg-Gly-Asp peptide mimetic) functionalized terpolymer and poly(methacrylic acid)- polysorbate 80-grafted starch-lipid NP	cross intact blood–brain barrier; enhance cellular uptake, cytotoxicity and drug delivery; selective targetability to human TNBC cells and murine macrophages	[93]
Doxorubicin and zymosan	NP complex composed of pegylated polyethylenimine and zymosan and Dox	enhance cellular uptake; induce apoptosis; induce secretion of proinflammatory cytokine; modify the biodistribution of DOX; reversed TAMs polarization from M2 to M1 phenotype; anti- angiogenic effect	[94]
Macrophage migration inhibitory factor-siRNA	glucan -based NPs	reduce tumor cell proliferation and enhance apoptosis; reduce the number of MIF at tumor; antitumor and anti-metastasis; increase CD4^+^ T cells infiltration	[95]
siCCR2	siCCR2-encapsulated cationic polymeric NP	block monocytes recruitment, reduce TAMs abundance in tumor tissues; reverse tumor immune suppression; enhance the antitumor effect of chemotherapy	[96]
VEGF siRNA (siVEGF) and PIGF siRNA (siPIGF)	PEG = MT/PC/siVEGF/siPIGF NPs a novel dual-stage pH-sensitive carrier composed of cationic polyethylene glycol (PEG) and mannose modified trimethyl chitosan conjugate (PEG = MT), and an anionic poly- (allylamine hydrochloride)-citraconic anhydride (PAH-Cit, PC)	inhibit the proliferation of BCs; reverse TAMs polarization from M2 to M1 phenotype; inhibit BC lung metastasis; anti-angiogenic effect	[97]
Hydrazinocurcumin	RR-11a-coupled liposomal NPs	suppress STAT3 activity; reverse TAMs polarization from M2 to M1 phenotype	[98]
Metformin	Hollow mesoporous manganese dioxide NPs coated with macrophage membranes	reverse TAMs polarization from M2 to M1 phenotype; target TAMs; suppress tumor growth	[99]
Zoledronic acid (ZOL)	Asn-Gly-Arg and PEG2000 modified liposomes	inhibit tumor growth; reduce TAMs; inhibit tumor angiogenesis	[100]

**Table 3:** TAM-mediated nanoparticles against breast cancer.

Payloads or Associated Agents	Nanocarrier	Function	References
Cytosine-phosphate-guanine (CpG)	3-aminopropyltriethoxysilane-modified Fe3O4 NPs	suppress the metastasis of BC to the lungs; increases infiltrating lymphocytes in tumors; stimulate humoral immune response	[106]
Macrophage Inflammatory Protein 3 Beta (MIP-3β)	NP complex composed of 1,2-Dioleoyl-3-trimethylammonium-propane, folic acid modified poly (ethylene glycol)-b-poly(ε-caprolactone) and methoxy poly (ethylene glycol)-poly(lactide)	activate CD8^+^ T-lymphocytes; induce DCs maturation; inhibit M2 polarization; suppress angiogenesis; suppress tumor growth and metastasis	[107]
Ganoderma lucidumpolysaccharide	Ganoderma lucidumpolysaccharide contained gold nanocomposites	induce DCs maturation; reverse the decline of CD4^+^ T cells and CD8^+^ T cells population; stimulate T cells proliferation; suppress tumor growth and metastasis	[108]
CD73 specific siRNA	CD73-specific siRNA-loaded chitosanlactate NPs	inhibit the expression of CD 73; reduce tumor growth rate; decrease Treg, MDSCs and TAMs, enhance CTL function; enhance the secretion of Th1 frequency and inflammatory cytokine network; antitumor and anti-metastasis;	[109]
Resiquimod CR848 and doxorubicin-hyaluronic acid conjugate (HA-DOX)	HA-DOX coated PHIS/R848 NPs	promote DCs maturation and activation; selective effects on breast cancer cells; regulate antitumor immune response by promoting infiltration of T cells and CTLs;	[110]
DOX,IL-2,IFN-γ	DC cell-derived nanovesicles	suppress tumor growth and metastasis; adsorb IFN-γ; enhance DCs mature; increase the infiltration of CTLs and activation of NK cells; increase the recruitment of CD45^+^immune cells and Ly6G^+^neutrophils	[111]
Doxorubicin	highly integrated mesoporous silica NPs	induce DCs maturation; improve drug accumulate in the tumor; induce anticancer immune response	[112]
Doxorubicin	Low-dose doxorubicin hydrochloride cancer cell membrane coated calcium carbonate NPs	induce ICD; CRT exposure; promote DCs maturation	[113]

**Table 4:** CAF-mediated nanoparticles against breast cancer.

Payloads or Associated Agents	Nanocarrier	Function	References
Doxorubicin	Cleavable amphiphilic peptide (containing a TGPA peptide sequence)-NPs	increase the effective drug concentration at the FAP-α-rich tumor sites; facilitate drug penetration through the stromal barrier; possess tumor targeting specificity	[124]
Telmisartan, Doxorubicin	Glycolipid polymer-based micelles composed of chitosan and stearic acid	Decrease activity of CAFs; inhibit CAFs secreted cytokines;	[125]
Gemcitabine,18β-glycyrrhetinic acid	NPs composed of dendrigraft poly-l-lysine, PEG-PCL, substrate peptide of MMP-2	regulate the chemoexposed TAFs; deliver drug to the deep region of tumor tissue	[126]
Paclitaxel	Liposome co-modified with acid-cleavable folic acid and dNP2 peptide	enhance the uptake and deep penetration in FR-positive tumor cells and FR-negative CAFs; exhibit synergistic TME targeting and blood brain barrier transmigration; accumulate in brain metastatic sites;	[127]
Sodium tanshinone IIA sulfonate and celastrol	A gold nanorod-anchored thermo- liposomal complex	normalize the tumor blood vessel; decrease the density of TAFs and collagen; regulate the secretion of cytokines	[128]
ZnF16Pc	ZnF16Pc-nanoparticle protein cage conjugated with a FAP- targeted single chain variable fragment	selectively eliminate CAFs; ECM destruction; suppress CXCL12 secretion; enhance CD8^+^ T cells infiltration	[129]
Pseudomonas exotoxin A/anisamide	Poly L lysine-based cationic carrier was coupled with the glutathione-sensitive disulfide bound vitamin E succinate and was covered with a sigma1 receptor and integrin αvβ3 receptor multitargeting	suppress angiogenesis; suppress tumor growth and metastasis; target elimination of CAFs;	[130]

## Data Availability

Not applicable.
